# Case report: Severe acute hepatitis in a 22-month-old Chinese boy with Omicron sub-variant BA.2.38

**DOI:** 10.3389/fpubh.2022.1012638

**Published:** 2022-11-24

**Authors:** Xinying Chen, Junbin Hong, Yuxia Li, Caixia An, Jianwen Guo, Jinghua Yang

**Affiliations:** ^1^Department of Pediatrics, The Second Affiliated Hospital of Guangzhou University of Chinese Medicine, Guangzhou, China; ^2^Xiaorong Luo's Renowned Expert Inheritance Studio, Guangdong Provincial Hospital of Chinese Medicine, Guangzhou, China; ^3^The Second Clinical Medical College, Guangzhou University of Chinese Medicine, Guangzhou, China; ^4^Department of Pediatrics, The Affiliated Hospital of Gansu University of Chinese Medicine, Lanzhou, China; ^5^Department of Pediatric Cardiology, Nephrology, Rheumatology and Immunology, Gansu Provincial Maternal and Child Health Hospital, Lanzhou, China; ^6^State Key Laboratory of Dampness Syndrome of Chinese Medicine, The Second Affiliated Hospital of Guangzhou University of Chinese Medicine, Guangzhou, China; ^7^Department of Neurology, The Second Affiliated Hospital of Guangzhou University of Chinese Medicine, Guangzhou, China

**Keywords:** severe acute hepatitis, SARS-CoV-2, Omicron variant, case report, pediatrics

## Abstract

The etiology of severe acute hepatitis (SAH) in children is various. We describe the first Chinese case of severe acute hepatitis in a 22-month-old boy with the mild illness of Omicron sub-variant BA.2.38. With the application of Compound Glycyrrhizin Injection (CGI), the patient gradually recovered from acute liver injury (ALI). This case highlights the possibility of severe ALI in children with the non-critical illness of SARS-CoV-2. The management of SAH associated with the pandemic presents challenges for clinicians, and follow-up is in need. The method of differential diagnosis using limited laboratory results is of great value to the clinicians.

## Introduction

The first outbreak of unexplained severe acute hepatitis (SAH) on April 5, 2022, has caused widespread concern worldwide. As of July 13, 2022, according to the World Health Organization (WHO), more than 1,000 cases have been reported all around the world, including 22 deaths in children (1). The cause of SAH is still complex and various. Based on the available evidence, it is currently believed that the underlying etiology may be infection of adenovirus (HAdV) and severe acute respiratory syndrome coronavirus 2 (SARS-CoV-2) (e.g., wild-type antigens, Delta or Omicron variant), as well as other unknown pathogens or co-infections (e.g., rhinovirus, parainfluenza, sapovirus, cytomegalovirus, norovirus, or enterovirus) (2–4).

Abnormal liver function tests were found in up to 47% of SARS-CoV-2 patients (5), with elevated transaminases common, especially in males and severe SARS-CoV-2 cases (6, 7). Transaminases like alanine transaminase (ALT) and aspartate transaminase (AST) were all predictors of disease severity and in-hospital mortality independently (5). However, SAH caused by SARS-CoV-2 was rare. However, we report a case of SAH in a young child infected with a sub-variant of Omicron BA.2, which, to our knowledge, is the first case of SAH associated with the SARS-CoV-2 Omicron variant reported in mainland China.

## Case presentation

A 22-month-old Chinese boy of Hui nationality who was previously healthy and had no history of liver injury tested positive for SARS-CoV-2 nucleic acid from a throat swab at the local hospital on July 19, 2022 (viral gene sequencing by the Center for Disease Control Laboratory, Lanzhou, Gansu, confirmed to be Omicron sub-variant BA.2.38). He had a fever with a peak of 38.9°C, and his body temperature returned to normal after oral ibuprofen suspension for only one time. However, he occasionally coughed, so he was transferred to Linxia Hospital of Traditional Chinese Medicine on July 21, 2022, and the fever and cough disappeared on their own. Blood routine showed neutropenia, and C-reactive protein (CRP) was at normal levels, which was related to SARS-CoV-2 infection. However, transaminase was significantly elevated (ALT>500 IU/L, AST>300 IU/L, Table 1) for two consecutive days, and then he was transferred to the No.2 People's Hospital of Lanzhou for further diagnosis and treatment. The patient's mother had a history of exposure to SARS-CoV-2 on July 13. He is the mother's second child, delivered by cesarean section at 36 weeks, and has not been vaccinated against the new coronavirus but has been vaccinated against BCG, hepatitis B, polio, DTP, measles, and meningitis. He was artificially fed when he was born and supplemented with complementary food after the 8 month. The milestones of growth and development were following age. His parents denied any family history, including any autoimmune or rheumatic disease.

**Table 1:** The findings of laboratory test.

**Date/Day of illness**	**2022/7/22** **(D3)**	**2022/7/23** **(D4)**	**2022/7/24** **(D5)**	**2022/7/25** **(D6)**	**2022/7/26** **(D7)**	**2022/7/27** **(D8)**	**2022/7/28** **(D9)**	**2022/7/29** **(D10)**	**2022/7/30** **(D11)**
WBCx10^∧^9/L(5.0-12.0)	3.53	4.63	/	15.7	15.2	/	13.2	/	8.7
NEUTx10^∧^9/L(2.0-7.0)	0.61	0.85	/	2.9	4.2	/	4.2	/	2.3
LYMx10^∧^9/L(5.00-12.00)	2.78	2.83	/	11.8	9.4	/	7.2	/	4.9
RBCx10^∧^12/L(4.00-5.50)	5.09	4.99	/	4.9	4.5	/	4	/	4.7
Hb, g/L(120-160)	136	136	/	127	118	/	105	/	123
PLTx10^∧^9/L(100-300)	216	188	/	258	265	/	127	/	329
CRP, mg/L(0.00-6.00)	<0.2	/	/	1.22	/	/	/	/	/
Ferritin, ng/mL(11.00-306.80)	/	129.9	/	148.4	/	/	/	/	2.27
ALT, U/L(9-50)	/	807.88	914.75	810	589	/	397	263	184
AST, U/L(15-40)	/	463.14	323.03	260	157	/	97	51	33
ALB, g/L(35.0-55.0)	/	43.67	42.36	47.3	45.1	/	/	43.6	44.9
GLB, g/L(20-30)	/	25.93	24.01	22.1	22.5	/	/	21.6	22.5
ALP, U/L(34-500)	/	376.57	391.91	/	/	/	/	280	271
GGT, U/L(10-60)	/	55.79	79.17	/	/	/	/	51	52
TBIL, μmol/L(5.1-29.5)	/	5.6	4.98	3	4.5	/	4.8	4.4	4.7
DBIL, μmol/L(0.0-6.8)	/	2.1	1.99	1.2	2	/	/	1.7	1.9
plasma ammonia, μmol/l(18-72)	/	/	/	69	/	/	/	/	/
TG, mmol/L(0.40-1.70)	/	1.73	/	1.6	/	/	/	/	/
TC, mmol/L(2.50-5.18)	/	4.51	/	4	/	/	/	/	/
Creatine kinase isoenzyme, U/L(0-25)	/	21.8	/	23	/	/	/	/	/
α-hydroxybutyrate dehydrogenase, U/L(72-182)	/	379.45	/	442	/	/	/	/	/
Na+, mmol/L(137.0-147.0)	/	138.52	/	142.9	140.6	/	/	138.9	142.7
K+, mmol/L(3.50-5.30)	/	4.59	/	5.25	4.51	/	/	4.73	4.62
Blood urea nitrogen, mmol/L(3.1-8.0)	/	5.01	/	5.3	/	/	/	/	/
Cr, μmol/L(57-97)	/	31.44	/	23	/	/	/	/	/
Activated partial thromboplastin time, seconds(25.0-40.0)	/	29.1	/	17.2	/	/	19.1	/	/
Prothrombin time, seconds(9.0-14.0)	/	8.2	/	10.5	/	/	10.9	/	/
INR	/	0.69	/	0.89	/	/	0.92	/	/
FIB, g/L(2.00-4.00)	/	2.64	/	2.57	/	/	3.08	/	/
DDi, μg/ml(0.00-0.55)	/	1.86	/	1.39	/	/	32.41	6.36	1.10
FDP, μg/ml(0.0-5.5)	/	/	/	5.4	/	/	/	/	/
AT-III%, (75-120)	/	/	/	111	/	/	/	/	/
(nasal swab)SARS-CoV-2-ORF1ab-CT, (>40)	/	/	/	/	36	26	29	/	32
(nasal swab)SARS-CoV-2-N-CT, (>40)	/	/	/	/	38	28	28	/	32
Other important findings	/	/	HAV-IgM(-)	HIVAb(-)	/	/	stool routine(-)	/	Reapted abdominal ultrasound(-)
			HEV-IgM(-)	TPAb(-)	/	/	/	/	EBV-IgM(-)
				HCVAb(-)	/	/	/	/	CMV-IgM(-)
				HBsAg/HBeAg/HBeAb/HBcAb(-)	/	/	/	/	HAV-IgM(-)
				HBsAb(-)	/	/	/	/	HEV-IgM(-)
				EBV-IgM(-)	/	/	/	/	/
				CMV-IgM(-)	/	/	/	/	/
				COVID-19-IgM(-)	/	/	/	/	/
				COVID-19-IgG(-)	/	/	/	/	/
				Abdominal ultrasound(-)	/			/	/

WBC, white blood cell; NEUT, Neutrophils; LYM, Lymphocytes; RBC, red blood cell; Hb, hemoglobin; PLT, platelets; CRP, C-reactive protein; ALT, alanine transaminase; AST, aspartate transaminase; ALB, albumin; GLB, globulin; ALP, alkaline phosphatase; GGT, gamma-glutamyl transferase; TBA, total bile acid; TG, triglycerides; TC, total cholesterol; TBIL, total bilirubin; DBIL, direct bilirubin; Na+, sodium; K+, potassium; Cr, creatinine; IgM, immunoglobin M; IgG, immunoglobin G; INR, international normalized ratio; FIB, fibrinogen; DDi, D-dimer; FDP, fibrinogen; AT-III, antiprothrombin III; HIV, human immunodeficiency virus; TP, treponema pallidum; HAV, Hepatitis A Virus; HCV, Hepatitis C virus; HBV, Hepatitis B virus; HEV, Hepatitis E Virus; EBV, Epstein-Barr virus; CMV, cytomegalovirus. SARS-CoV-2-ORF1ab, cycle threshold (CT) of open reading frame 1ab of severe acute respiratory syndrome coronavirus 2; SARS-CoV-2-N, CT of nucleocapsid protein of severe acute respiratory syndrome coronavirus 2; COVID-19, the new coronavirus disease 2019; HBsAg, hepatitis B surface antigen; HBeAg, hepatitis B e-antigen; HBeAb, hepatitis B e-antibody; HBcAb, hepatitis B core antibody; HBsAb, hepatitis B surface antibody.

On admission, the child had a slightly poor appetite, without fever, diarrhea, jaundice, hypoxia, or other discomforts. Physical examination showed that, except for red lips, his vital signs were within the normal range. No dry or wet rales were found in the lungs. The liver was palpated 1 cm below the rib edge (consistent with age), and the spleen was not palpated. He was diagnosed with the mild illness of SARS-CoV-2 infection. Laboratory tests revealed severe hypertransaminasemia (ALT, 810 IU/L; AST, 260 IU/L) without elevated bilirubin. Also, plasma ammonia and coagulation function were in the normal range, which did not support the diagnosis of liver failure. Blood routine showed that he had elevated white blood cells, mainly lymphocytes, but no abnormality in acute phase reactants such as CRP and ferritin, as well as biochemical indicators.

Further examination ruled out the possibility of abnormal liver function caused by hepatitis A, hepatitis B, hepatitis C, hepatitis E, cytomegalovirus, Epstein-Barr virus, human immunodeficiency virus, and Treponema pallidum infection. Abdominal ultrasound (on Day 6 and 11 of the disease) ruled out organic liver disease.

We also considered non-infectious factors in the case, such as inherited metabolic liver disease (IMLD) and autoimmune hepatitis (AIH). IMLD is a class of diseases that impair the synthesis and decomposition of metabolites due to genetic defects, such as the repeated occurrence of metabolic abnormalities such as glucose, lipids, amino acids, bilirubin, and bile acids, which usually lead to Childhood growth retardation. Besides, family history is a high-risk factor. However, laboratory results showed that the participant's blood glucose, lipids, protein, plasma ammonia, and bilirubin were all within normal ranges, and he was not showing signs of growth retardation, which did not support the diagnosis of IMLD. AIH is a chronic, progressive liver disease mediated by immune inflammation, characterized by circulating autoantibodies and elevated serum globulin levels, and glucocorticoids and other immunosuppressive agents are used to induce remission. Despite the lack of testing for autoantibodies, the serum globulin levels in the patient did not elevate several times, and the transaminases gradually improved without corticosteroids or other immunosuppressive therapy, which indicates he was not suffering from AIH.

**Figure 1:**
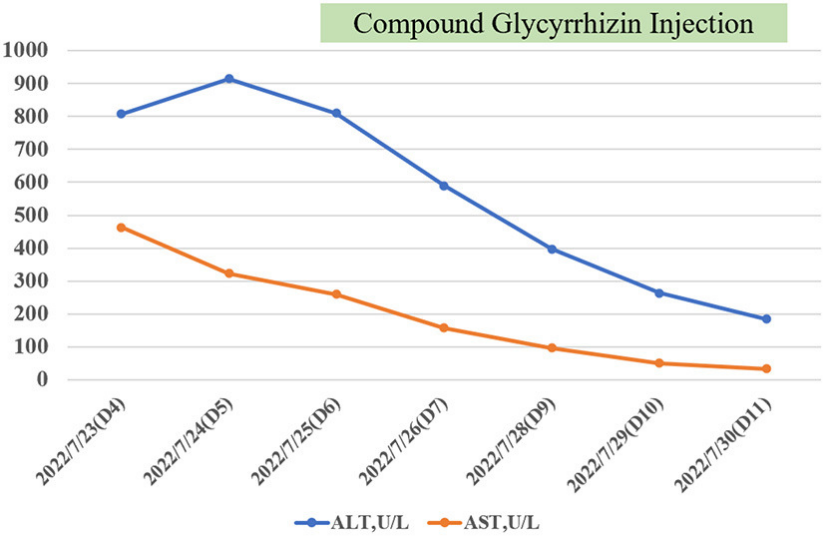
The trend of transaminase. With the use of compound glycyrrhizin injection (CGI), the patient gradually recovered from acute liver injury.

Referring to the WHO working case definition for SAH, the child was diagnosed with SAH. Compound glycyrrhizin was administered intravenously due to acute liver injury (ALI). Subsequent results suggested a gradual improvement in liver damage (Table 1; Figure 1). On the ninth day after the disease diagnosis, the D-dimer was significantly elevated but asymptomatic, and it returned to a normal range without specific medication. The patient is currently in good condition.

## Discussion

We demonstrated a case of definite SARS-CoV-2 infection with SAH, but the child had mild symptoms of SARS-CoV-2 infection, no jaundice or liver failure, and did not require respiratory support or intensive care. According to the working case definition for SAH in children by WHO and other organizations (8, 9), there are some differences. However, it is consistent that children under 16 years old (sometimes 10 years old) can be diagnosed with severe acute hepatitis when the transaminase (ALT or AST) is significantly elevated (>500U/L). Therefore, the diagnosis of SAH in the child was unquestionable. To our knowledge, this is the first pediatric case of SAH with Omicron sub-variant BA.2.38 reported in mainland China.

The clinical symptoms of SARS-CoV-2 infection in children are diverse. Different from adults, children usually show only milder symptoms, but in some cases, children may develop a multisystem inflammatory syndrome (MIS) (10, 11). It affects the respiratory tract, gastrointestinal tract, liver and other extrapulmonary organs. Fever and cough are the most common clinical symptoms (12), and the incidence of ALI is not low (13–15). ALI in children often indicates severe disease (16) and we should take it seriously.

Currently, the pathogenesis of SARS-CoV-2-related ALI has not been fully clarified, mainly divided into direct and indirect injury. On the one hand, liver biopsy pathology showed that SARS-CoV-2 could directly influence liver cells and result in cell apoptosis (17). In addition, SARS-CoV-2 also directly infects bile duct cells and causes bile duct dysfunction (18). Bile duct cells can specifically express angiotensin-converting enzyme 2 (ACE2), the cellular receptor for SARS-CoV-2 entry (19). On the other hand, SARS-CoV-2 infection can cause the release of a large number of pro-inflammatory factors (such as interleukin IL-6, etc.), induce cytokine storm syndrome, and cause immune-mediated liver damage (20). Under systemic stress, there is a compensatory decrease in peripheral and splanchnic blood flow, leading to a decrease in hepatic blood flow, which leads to hepatocyte hypoxia and is one of the possible causes of ALI (20).

Among children infected with SARS-CoV-2, the majority had mild to moderate ALI, which was usually associated with a more extended hospital stay and a more severe clinical course, broadly consistent with ALI in adults (13–15). ALI is usually fully recoverable (21), but in children with MIS and ALI, more than half of the children had persistent liver function damage 1 month after discharge (21), suggesting that follow-up in children with ALI is necessary.

Severe ALI (SALI), such as SAH or acute liver failure, is relatively rare. Patients with SALI may rapidly progress to acute liver failure with hepatic encephalopathy, coagulopathy, and refractory hyperammonemia, leading to death in 10-month-old and 11-year-old boys, respectively (22, 23). In addition, two infants younger than six months developed liver failure rapidly and required liver transplantation (24). However, recent evidence suggests that SAH may be temporary. One article reported four cases of SAH associated with SARS-CoV-2, ranging in age from 6 months to 16 years, with no deaths, 1 of whom required tracheal intubation during hospitalization. After discharge, 2 patients recovered a month later, while the rest recovered 2 and 8 months later, respectively (25). In addition, there was a 30-day-old and a 5-year-old patient with SAH but no liver failure and no need for intensive care (26, 27), which is consistent with our participant. Therefore, the prognosis of SAH is not always optimistic.

ALI caused by SARS-CoV-2 brings challenges to clinical work, and clinicians should pay attention. Long-term follow-up is necessary, especially in children with SALI, before the liver function returns to normal. Further research on markers of early SAH or liver failure is also required for early intervention and prognosis improvement as much as possible.

There are significant challenges that remain in the diagnosis and management of SAH cases during the pandemic. The available testing materials are limited, especially when they occur in poor-resource areas (9). Similarly, our case had some limitations due to the lack of some specific laboratory findings, which was a real dilemma in clinical work. Nevertheless, we have tried our best to make a differential diagnosis from clinical symptoms, signs, risk factors, and other aspects. In our case, the method of differential diagnosis using limited laboratory results is of great value to the clinician.

## Conclusion

We report a child of SAH associated with SARS-CoV-2 Omicron variant infection in the Chinese mainland, which highlights the possibility of SALI in children with a non-critically illness of the pandemic. The management of SAH associated with SARS-CoV-2 presents challenges for clinicians. Early identification, early diagnosis, early treatment, and long-term follow-up are crucial. More research is in need in the future.

## Data availability statement

The original contributions presented in the study are included in the article/supplementary material, further inquiries can be directed to the corresponding authors.

## Ethics statement

The studies involving human participants were reviewed and approved by Ethics Committee of Guangdong Provincial Hospital of Chinese Medicine. Written informed consent to participate in this study was provided by the participants' legal guardian/next of kin. Written informed consent was obtained from the participant/participant's legal guardian/next of kin for the publication of this case report.

## Author contributions

XC, JY, and JG conceptualized and designed the study. XC drafted the initial manuscript and revised the manuscript. JH, YL, and CA designed the data collection instruments, coordinated, and supervised data collection. JY and JG reviewed and critically revised the manuscript. All authors approved the final manuscript as submitted and agree to be accountable for all aspects of the work.

## Funding

This study was supported by State Administration of Traditional Chinese Medicine, an urgent project for Chinese Medicine on Novel Coronavirus Pneumonia (No. 2022ZYLCYJ07-3), and Xiaorong Luo's Renowned Expert Inheritance Studio (No. 14GG2X02).

## Conflict of interest

The authors declare that the research was conducted in the absence of any commercial or financial relationships that could be construed as a potential conflict of interest.

## Publisher's note

All claims expressed in this article are solely those of the authors and do not necessarily represent those of their affiliated organizations, or those of the publisher, the editors and the reviewers. Any product that may be evaluated in this article, or claim that may be made by its manufacturer, is not guaranteed or endorsed by the publisher.
